# Implications of landscape genetics and connectivity of snow leopard in the Nepalese Himalayas for its conservation

**DOI:** 10.1038/s41598-020-76912-7

**Published:** 2020-11-16

**Authors:** Bikram Shrestha, Pavel Kindlmann

**Affiliations:** 1 Department of Biodiversity Research, Global Change Research Institute CAS, Bělidla 986/4a, 603 00 Brno, Czech Republic; 2 Institute for Environmental Studies, Faculty of Science, Charles University, Benátská 2, 128 01 Prague 2, Czech Republic

**Keywords:** Biodiversity, Conservation biology

## Abstract

The snow leopard is one of the most endangered large mammals. Its population, already low, is declining, most likely due to the consequences of human activity, including a reduction in the size and number of suitable habitats. With climate change, habitat loss may escalate, because of an upward shift in the tree line and concomitant loss of the alpine zone, where the snow leopard lives. Migration between suitable areas, therefore, is important because a decline in abundance in these areas may result in inbreeding, fragmentation of populations, reduction in genetic variation due to habitat fragmentation, loss of connectivity, bottlenecks or genetic drift. Here we use our data collected in Nepal to determine the areas suitable for snow leopards, by using habitat suitability maps, and describe the genetic structure of the snow leopard within and between these areas. We also determine the influence of landscape features on the genetic structure of its populations and reveal corridors connecting suitable areas. We conclude that it is necessary to protect these natural corridors to maintain the possibility of snow leopards’ migration between suitable areas, which will enable gene flow between the diminishing populations and thus maintain a viable metapopulation of snow leopards.

## Introduction

The snow leopard (*Panthera uncia*) is commonly seen as a flagship species of the high altitude ecosystems^1^. It is the smallest species within the genus *Panthera*^1^. It preys mainly on wild ungulates (blue sheep, *Pseudois nayaur*, Siberian ibex, Capra *sibirica*, Himalayan tahr, *Hemitragus jemlahicus,* argali, *Ovis ammon*) and domestic animals. Small mammals, such as marmots (*Marmot* spp.) and lagomorphs serve as its supplementary food^2–6^.

Snow leopard lives in alpine and sub-alpine meadows of central Asia in the altitudes of 2500 to 5800 m^1,7,8^. It is therefore well adapted to low temperatures^1^. One can find it typically in a steep terrain, where cliffs, ridges, gullies and rocky outcrops are common^7,8^.

It is estimated that there exist 2710 to 3386 mature individuals of snow leopard in the world^9^. Recently, its categorization in the IUCN Red List^9^ has been downgraded from endangered to vulnerable. However, its population size has declined by 10% over the last 3 generations and this species now occupies only 27% of its vast potential range^1,9^. Therefore, debates arose regarding its down listing in the IUCN Red List. Some people argue that it was based on few samples in the prime habitat of its distribution range, which could have attributed to overestimation of its abundance^10^. The low and declining population size of snow leopard is most likely a consequence of human expansion in these areas, which entailed a loss of suitable habitat and of wild prey for snow leopard^1,8,9^. A straightforward consequence of this was more frequent killing of livestock by snow leopard^1,8,9,11^. In return, people resorted to retaliatory killings and poaching of snow leopard^1,9,10^. Other causes of the decline in snow leopard numbers include diseases and climate change^1,9,12–14^. With the latter, a considerable part of snow leopard’s habitat may be lost because of an upward shift in the tree line and concomitant loss of the alpine zone^12^.

Snow leopard tends to inhabit areas with a particular type of habitat. Within these areas, adult snow leopards generally occur solitarily^7,15^. Between such areas, snow leopards are recorded only occasionally and are then moving between suitable areas. Based on data on its occurrence, a good and reliable map of the snow leopard distribution and migration can be produced by using habitat suitability models. Such maps act as the main components for good management plans. Migration between suitable areas may result in inbreeding^16^, fragmentation of populations^17^, reduction in genetic variation due to habitat fragmentation, loss of connectivity^18^, bottlenecks or genetic drift^19^. Conservation genetics then reveals the key factors, which may cause snow leopard extinction, in such situations^20^. Thus both population genetics and habitat suitability models are crucial for designing proper management plans for its conservation^13,21,22^.

After the snow leopard-specific primers have been developed that enable to amplify mitochondrial DNA and polymorphic microsatellite loci^23,24^, it became popular to identify many characteristics based on genetic studies. This included determination of species, sex, and individuals, and estimation of population size and density^25–30^, determination of genetic diversity at microsatellite loci^24,28^, identification of phylogeography and genetic structure in the global range^31^ etc. However, studying only population genetic structure by itself is not sufficient. In Nepal, only one study^28^ analysed descriptive genetic diversity, and none exists on spatial population genetic structure based on Bayesian clustering, which is crucial for management plans aiming at snow leopard long-term persistence^20^.

The decline in the area of suitable habitat due to human activities and global change will make migration between such areas increasingly difficult^1,8,9^. To assure the future of snow leopards, it is necessary to safeguard the survival of this species as a metapopulation, which includes maintaining the connectivity between such areas^13,21,22^, as only this will preserve its genetic variability^31^. Although some studies on habitat suitability were performed in Nepal^32–34^ and in Tibet^35,36^, the connectivity between them has never been analysed.

We also need to know, how the populations differ genetically within and between such areas, in order to determine, which areas host rare genotypes and are therefore especially worthy of preservation^17–19^. Therefore, it is necessary to determine (i) the position of the corridors connecting individual areas and (ii) the genetic structure of the snow leopard metapopulation.

Here we aim to identify suitable habitats for snow leopards in Nepal, connectivity between them by finding realistic corridors connecting them, and describe the genetic structure of the snow leopard within and between the areas studied. We shall be using habitat suitability maps for this reason.

We had a good reason for choosing Nepal as a model country: in Nepal, mountain communities are strongly involved in snow leopard conservation^1^. Therefore, we expect that our suggestions for the maintenance of migration corridors and areas suitable for snow leopards will be well received there.

## Results

### Spatial structure of the population

**Figure 1:**
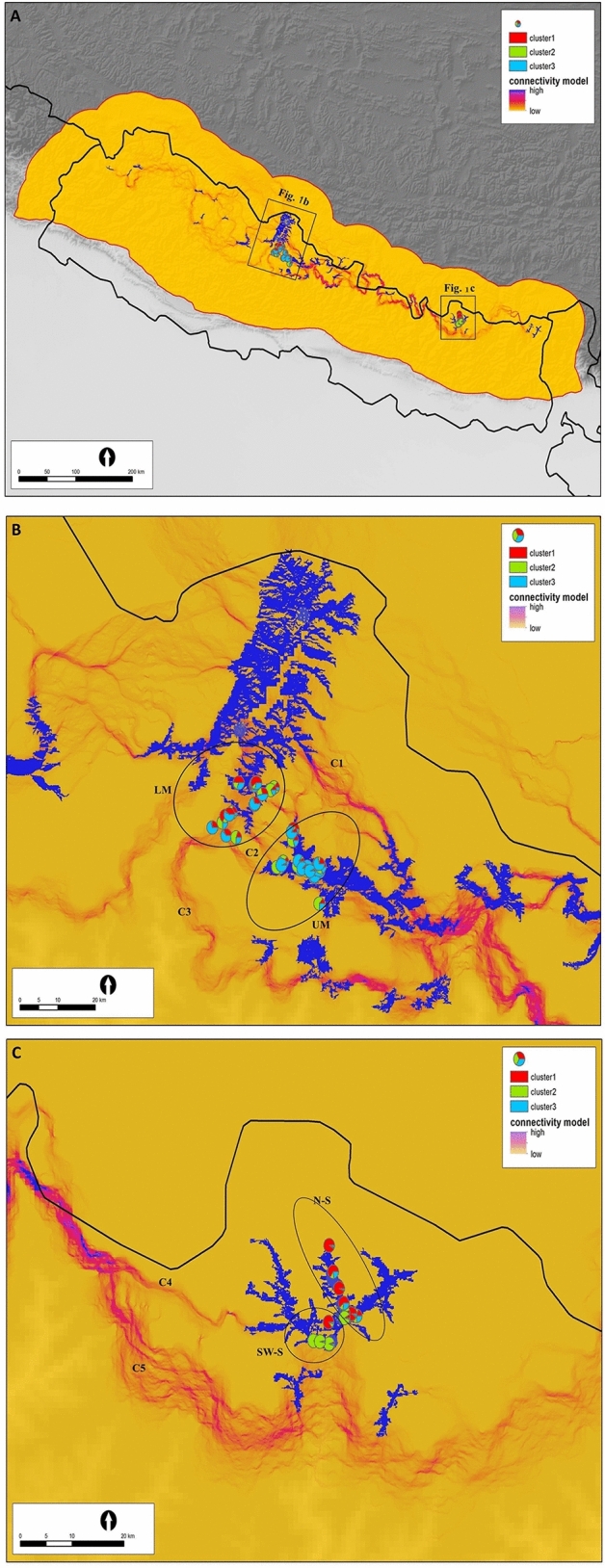
Maps showing: (i) suitable habitats (in blue; habitats with suitability index > 0.5 and area of at least 10 km^2^—see Material an Methods: Habitat suitability and connectivity analyses for definition); (ii) areas where signs of snow leopard were found (ellipses), (iii) exact locations of the signs (pie charts) and (iv) possible corridors (numbered as C1, C2, …, C5; in red). Colour segments in the pie charts of (iii) are Structure q values according to Bayesian clustering of the genetic samples using K = 3 (see the three population clusters in the inset). (**A**) whole Nepal and part of Tibet; (**B**) Lower Mustang (LM) and Upper Manang (UM) in the Annapurna Conservation Area; (**C**) North Sagarmatha (N-S) and South-West Sagarmatha (SW-S) in the Sagarmatha National Park.

Out of the 268 samples of scat, hairs and urine collected in the three areas studied (Fig. 1), 128 were identified as snow leopard (124 scat and 4 hair samples) based on a mitochondrial DNA species identification.

No microsatellite loci were identified in the twenty snow leopard samples, so they were not included in the following analyses. Thus, finally we had 108 microsatellite samples of snow leopard. After data quality filtering, 63 microsatellite genotypes were obtained, corresponding to 22 individuals according to the identity analysis. All loci were free of errors due to large allele drop out and stuttering, but two loci were suspect because of the presence of null alleles with estimated percentages of 20.27% and 14.45%, respectively. These two loci had higher proportion of homozygotes than expected according to the allele frequency.

Bayesian clustering in Structure clearly attributes the samples from Sagarmatha to spatially separated clusters (Figs. 1A and 1C): one in the northern (N-S) and the other in the south-western (SW-S) part. Samples from Upper Manang (UM) and Lower Mustang (LM) are much more mixed (Figs. 1A and 1B). According to the ΔK parameter in Structure, K = 3 is the most probable number of clusters. The conventional Structure plot can be found in Benesova^37^.

Genetic differences between the three areas studied are analysed in Table 1. Observed heterozygosity is moderate and ranges here between 0.54 and 0.61. Based on fixation index (genetic distance), there is less genetic differentiation in LM and UM, moderate genetic differentiation in SW-S while no differentiation in N-S, however overall there is significance genetic differentiation. See Benesova^37^ for details.

**Table 1:** Genetic characteristics recorded for the snow leopards studied. *Source*: Benesova^37^

Area	Na	Ne	Ho	He	F
Upper Manang (UM) and Lower Mustang (LM)	9	3	0.542	0.632	0.141
South-West Sagarmatha (SW-S)	9	3.609	0.556	0.666	0.165
North Sagarmatha (N-S)	5.167	2.715	0.61	0.533	−0.144
Overall	8.167	3.402	0.475	0.642	0.276
SE	0.654	0.601	0.072	0.076	0.039

Na = No. of different alleles, Ne = No. of effective alleles = 1 / (Sum pi^2), Ho = Observed heterozygosity = No. of Hets / N, He = Expected heterozygosity = 1—Sum pi^2, F = Fixation index = (He—Ho) / He = 1—(Ho / He), where pi is the frequency of the i-th allele in the population & Sum pi^2, which is the sum of the squared population allele frequencies. Samples from UM and LM were merged for this analysis, because there are much more genetic similarities.

### Characteristics of the range and results of the connectivity analysis

**Figure 2:**
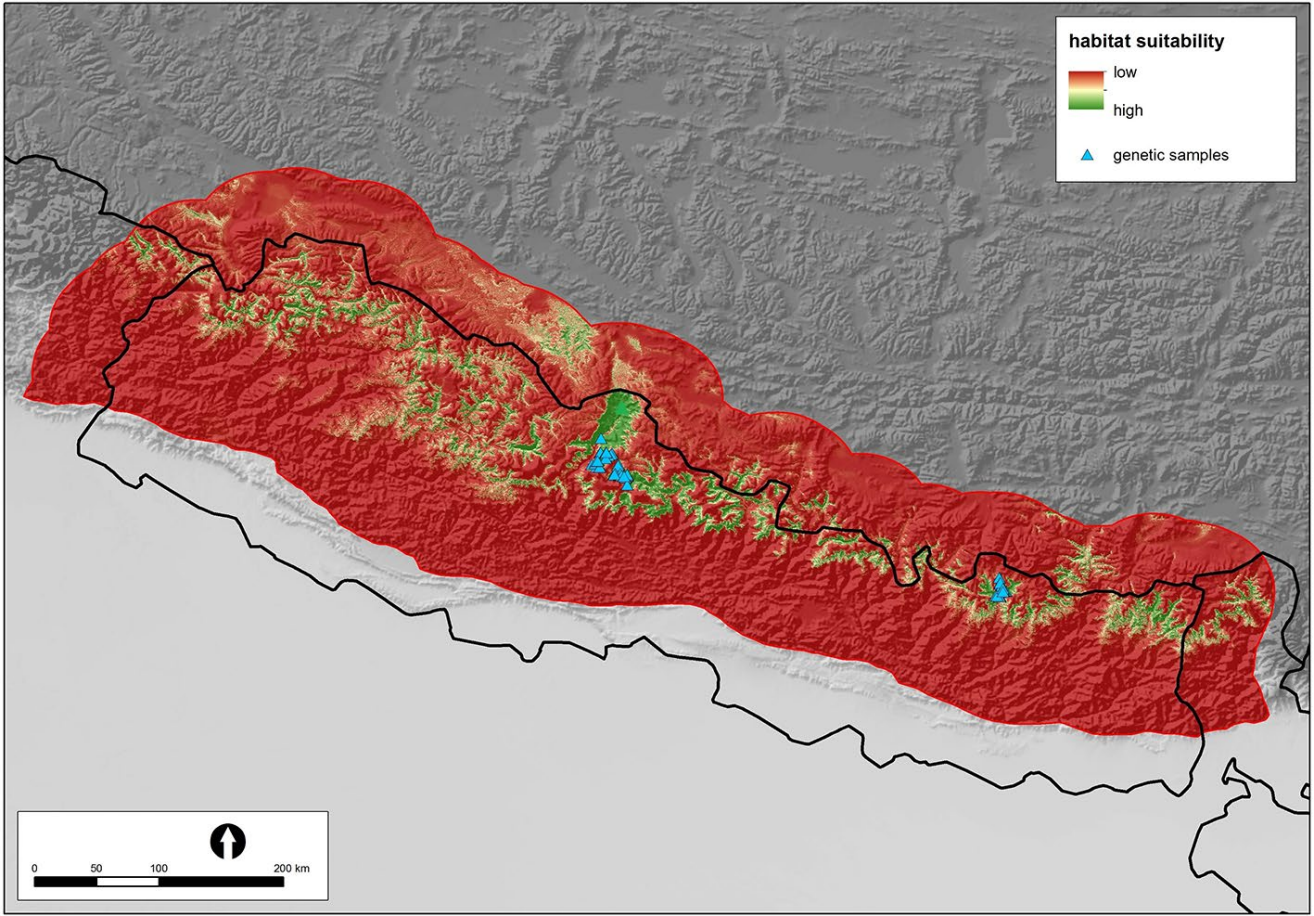
Map of Nepal showing the distribution of suitable habitat for snow leopard predicted by the model. Different colours indicate the probability of occurrence of snow leopard (see inset). The most suitable areas are in green. Locations, where we collected samples, are indicated by blue triangles.

The habitat suitability map is shown in Fig. 2. The suitable areas are indicated by yellow – green colour in this figure.

**Figure 3:**
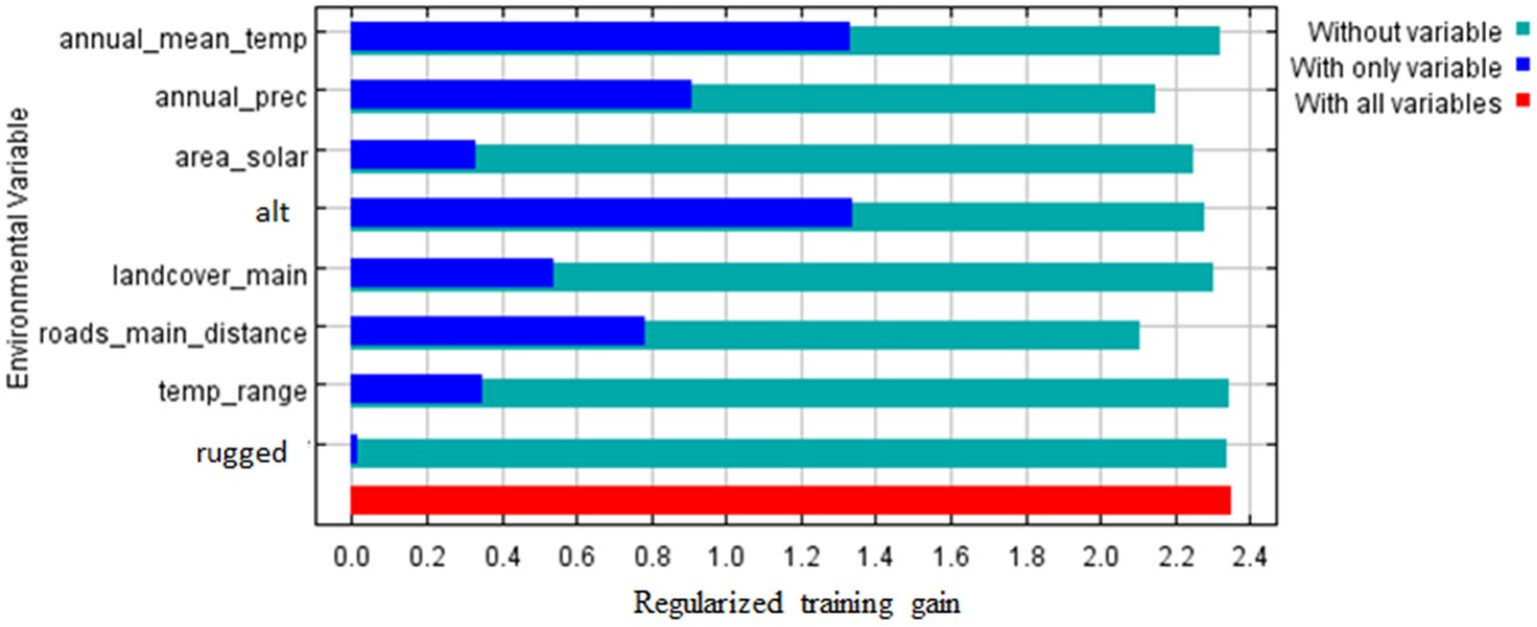
Relative importance of predictor variables of snow leopard presence, calculated by the jackknife test^38^. The length of the dark-blue bar indicates the size of the impact of selected variable, while the length of light-blue bar is showing how much of the explanatory power of the model would be lost if the corresponding factor were excluded from the analysis.

In the Maxent model, the receiver operating characteristic (ROC)^38,39^ results of the AUC value^38,39^ was high for the training data (0.974), indicating that the predictions were excellent and potentially useful. A close look at the Maxent’s jackknife test of variable importance^38,39^ (Fig. 3) shows that:

(i) The most important environmental variables defining the distribution of snow leopard within the area studied were altitude (alt), annual mean temperature (annual_mean_temp), annual precipitation (annual_prec), and distance from roads (roads_main_distance).
(ii) Some other factors also seem to play a role: land cover (landcover_main), solar radiation (area_solar) and mean diurnal range of temperature (temp_range).
(iii) Omitting the distance from roads from the model causes the largest loss of the explanatory power of the model, compared with omitting any of the remaining variables.
(iv) Ruggedness index (rugged) by itself is not useful for estimating the distribution of snow leopard.

**Figure 4:**
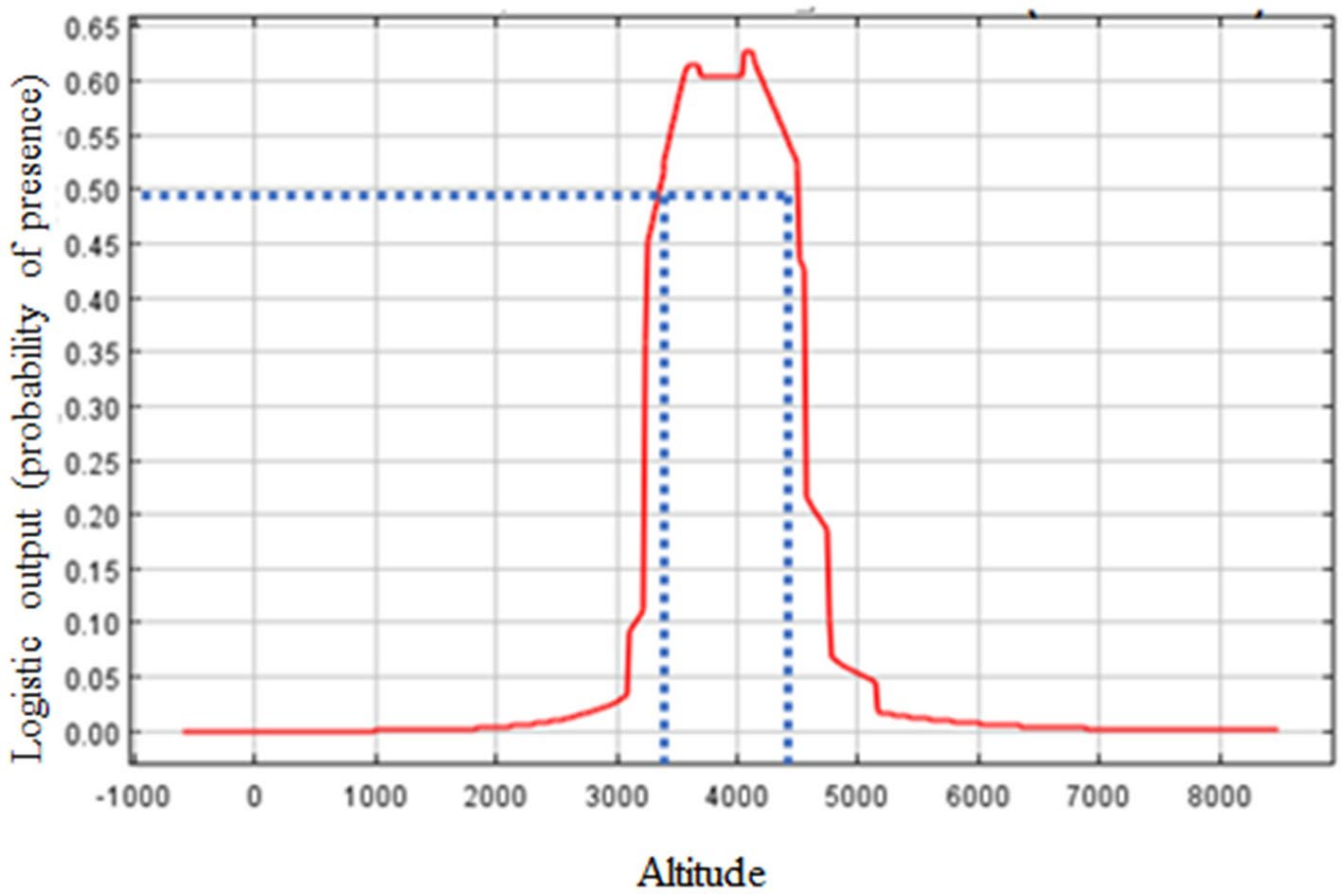
Response curve of *Panthera uncia* to altitude.

Spatial distribution of the most suitable habitats is a relatively narrow and compact belt at altitudes between ca 3500 and 4500 m, when 50% is considered as a threshold for the suitability habitat index (Fig. 4). This belt is widest in regions in western and central Nepal (e.g. in Mustang – see Fig. 1) and narrow in eastern Nepal (Fig. 2). The latter is therefore more vulnerable to fragmentation. Patches of the most suitable habitat are typically southern slopes at an altitude of around 4000 m with a relatively cold and dry climate, shrubs, rocks and open grassland.

### Landscape genetic approach and data integration

Habitat connectivity was analysed in order to produce resistance distances between particular localities, for which there were genetic samples and a voltage map indicating the corridors that might be used for dispersal or migration (Fig. 1). Voltage maps reveal regional differences in habitat connectivity, in which highly suitable areas for snow leopards in Central and Eastern Nepal (Fig. 1A) are interconnected by rather narrow corridors, which are very vulnerable, because they contain bottlenecks. In Western Nepal (LM, UM, Fig. 1B) the spatial pattern differs from that in eastern localities, as there are many alternative routes and corridors for migration or dispersal.

There are three possible corridors connecting the study areas LM and UM (Fig. 1B):

- (C1) – which starts in the Nar and Phu valleys, about 40 km east of Manang, continues north-west and reaches Mustang north of the LM study area;
- (C2) – connects UM and LM directly, but crosses the mountain ridge stretching from Khatung Kang peak (6484 m) to Tilicho lake (4919 m);
- (C3) – the southern link between Lamjung and Mustang.

There are two possible corridors connecting the western areas (LM, UM) with the eastern one (SNP): (C4) and (C5) (Fig. 1C), which in the westward direction merge and pass through three protected areas: Gaurishankar Conservation Area (GCA), Langtang National Park (LNP) and Manaslu Conservation Area (MCA). However, because of high mountain barrier between these protected areas, the corridor goes partially through the Tibetan Qomolangma National Nature Reserve.

For the whole study area, comparing FST using geographic distances revealed a weak effect of IBD (r = 0.22155; Z = 264,714,021.85418; p < 0.01). A weak effect of IBR is also present (r = 0.15785; Z = 455,729.95660; p < 0.001) as was revealed by comparing FST with resistance distances.

## Discussion

We revealed that annual mean temperature, altitude, annual precipitation and distance from roads were the four main factors influencing habitat suitability for snow leopard in Nepal. Bai et al.^36^ suggested five main factors (driest quarter, ruggedness, altitude, maximum temperature of the warmest month, and annual mean temperature) in the Qomolangma National Nature Reserve, Li et al.^35^ reported two main factors: annual mean temperature and ruggedness in Sanjiangyuan National Nature Reserve, and Aryal et al.^34^ reported only one factor: annual mean temperature. Thus, all the studies just mentioned stress climatic factors, especially annual mean temperature and altitude as the main determinants of snow leopard presence.

Our results differ from other ones, as regards ruggedness, which did not have an effect on snow leopard presence in our data. However, ruggedness is usually considered as profitable for snow leopard, as it provides shelter and possibilities to escape^7,15^. It is possible that our results are an artefact of the Maxent modelling, because the scale of the ruggedness layer available may not have been sufficiently fine. The preferred altitudinal range of snow leopard is between ca 3500 and 4500 m. This may be because this approximates the area above the treeline and below the line of maximum plant growth (alpine zone), where the snow leopard’s main prey grazes.

According to the genetic results, the gene flow between Manang and Mustang is sufficient to prevent the effects of genetic drift in these two regions. The results of the connectivity analysis reveal three possible ways by which snow leopards can move between Manang and Mustang: corridors (C1), (C2) and (C3) – see Fig. 1B. Corridor (C1) does not connect the study sites directly, but could facilitate gene flow between them. It is unlikely that corridor (C2) is used, because it involves climbing great heights: Khatung Kang peak (6484 m) and Tilicho Tal lake (4919 m). Dispersing individuals are thought to be less habitat-specific than residents^40^. However, our camera trap studies indicate the opposite: we photographically captured 5–8 snow leopards at each of the sites (both in UM an LM), but never photographed the same individual at both sites (Shrestha et al. in press).

Bayesian clustering in Structure showed that some samples in the Lower Mustang (LM) area were genetically close to those in the south-western part of the Sagarmatha (SW-S), which is approximately 300 km apart. The habitat between them contains many snow leopard habitat patches, as shown in Fig. 1A and three snow leopard populations—those in GCA, LNP and MCA. This means that the study sites are potentially connected via a series of “stepping stones” that could facilitate dispersal among local populations. Thus, there appears to be a certain degree of gene flow between these areas, indicating that a fraction of the population disperses over long distances, which is typical of large carnivores^40^. The average daily distance covered by snow leopards was thought to be 1–7 km^7^. However, recent satellite GPS-based data show that snow leopard can cover even 27–40 km per day^15^ and that the average home range is 200–500 km^15,41^. Analyses in Circuitscape reveal two access paths to Sagarmatha, one southern and one western path. According to the results of the cluster analysis there should also be a northern path, probably via the Tibetan plateau, as the samples from northern Sagarmatha (SNP) cluster with those from northern Mustang (LM). Lovari et al.^26^ suggest that Sagarmatha was recolonized via the Nangpa Pass from the north, a path not considered in the present study, because we only focused on Nepal.

The results of the landscape genetic analysis indicate a certain degree of gene flow between the two areas studied (LM & UM vs. N-S & SW-S). However, the movement of snow leopards between these areas may be difficult, because (i) the corridor connecting these areas is often narrow (to the west from merging of corridors C4 and C5, suitable habitats consist of narrow strips around the peaks of the mountains, as indicated by the IBD and IBR analyses) – see Fig. 1A and 1C – and (ii) although there are several snow leopard populations on the way (in GCA, LNP and MCA), human presence there may make the snow leopard movement difficult. The corridor connecting LM & UM with N-S & SW-S goes partially through Tibet: through the Qomolangma Nature Reserve in China. However, there are no available stepping-stones there either, as only three snow leopard populations, completely isolated by high mountains, are reported from here^36^ and none of these populations lives close to this corridor.

Because of the discrepancy described in the previous paragraph, it is essential to check the functionality of these corridors and identify others to ensure snow leopard's mobility among suitable areas as soon as possible. If this will not be done, survival of its metapopulation may be jeopardized because of impossibility of between-populations movement.

Both the connectivity and genetic analyses indicate that the populations in N-S and SW-S are genetically isolated from each other. A plausible reason for this is that each of these sub-populations was established from a different source population, when snow leopard was recently re-established in Sagarmatha after more than 40 years of absence^26,42^. The gene flow between these sites might have been limited by the barrier effect of two large rivers (Dudh Khosi and Imja) separating them. Also, the only possible corridor between these sub-populations is narrow and frequently used by tourists on the route to Namche Bazaar. There are hundreds of people on the route during the tourist season^43^, which may be a major factor preventing using it by snow leopard^44^. Thus, because of the short time since the re-establishment and limited gene flow, the genetic differences between the N-S and SW-S might have been preserved.

Similarly to other studies, even here the sightings of snow leopards were very rare. This rarity of sightings in the wild suggests that these animals tend to avoid humans^7,44^, in accord with observed effects of human activities on habitat use by other large predators such as grizzly bears, wolves, and tigers^44–48^. Generally, predators avoid large or frequented roads and trails, especially in areas where hunting or harassment is common^44,45,47,49,50^. Thus, humans may be a substantial determinant of snow leopard presence, in accord with results of our study. The spatial distribution of snow leopard and its prey overlaps with human-modified areas in Nepal. Human development and anthropogenic disturbance, like light pollution or human-associated sounds, change large carnivores’ behaviour^51,52^ and jeopardize their survival.

To summarize: we show that the genetic structure of snow leopard populations is mainly influenced by the proximity of people and trekking routes used by tourists, which pose a barrier to the dispersal of snow leopards. Topography of the terrain also plays a major role, as it determines the occurrence of suitable habitat for snow leopards in Nepal.

## Conclusion

The main message of this paper is the delimitation of the areas with suitable habitat for snow leopards in Nepal, which is presented in Fig. 2, and identification of the main corridors connecting these areas (corridors (C1) – (C5) in Fig. 1). We also determined the influence of landscape on the genetic structure of the population. Isolation could be attributed to either geographic barriers or human disturbances. It is necessary to protect these natural corridors, so that snow leopards can migrate between areas of suitable habitats. This is essential for the gene flow between the diminishing or even disappearing populations and for the survival of a viable population of this rare species. However, maintenance of these corridors may also help to preserve habitat connectivity for other species such as wolves, lynx, golden jackal and red foxes detected in camera traps and carnivore DNA sequence^53,54^.

## Materials and methods

### Study areas

In Nepal, snow leopards are found in the northern Himalayas, in mainly five areas: Shey Phuksundo National Park, Annapurna Conservation Area, Manaslu Conservation Area, Sagarmatha National Park, Kanchenjunga Conservation Area. The total population of snow leopards in Nepal is about 400 individuals^33^.

This study was carried out in the following four study sites:

1. Jomsom, Muktinath and Jhong valleys in Lower Mustang (called LM hereafter, covering ca 100 km^2^),
2. Proper Manang, Khangsar and Tanki Manang valleys in Upper Manang (called UM hereafter, covering ca. 105 km^2^),
3. Gokyo and Phortse valleys in the north of the Sagarmatha National Park (called N-S hereafter, covering ca. 50 km^2^).
4. Namche, and Thame valleys in the south-west of the Sagarmatha National Park (called SW-S hereafter, covering ca. 50 km^2^).

Sites LM and UM are in the Annapurna Conservation Area (ACA), sites N-S and SW-S are in the Sagarmatha National Park (SNP). All four sites were described in our previous paper^4^ and therefore we do not repeat their description here. In the Thame valley, no snow leopards were detected in either camera traps or scat samples.

### Scat sampling and snow leopard species identification

In total, 268 putative snow leopard samples (261 samples of faeces, 6 of hair and one of urine) were collected by using Snow Leopard Information Management System (SLIMS^55^) during 2014–2016. DNA from the samples was extracted and a ~ 148 bp segment of mitochondrial cytochrome-b was PCR-amplified using the primers CYTB-SCT-PUN-R’ and CYTB-SCT-PUN-F’^56^ for the species identification. The camera trap survey, scat sampling and snow leopard species identification methods were designed for studying abundance of snow leopard and its prey and diet of the former—see Shrestha et al.^4^

### Leopard Sex ID PCR

The sex identification of the verified snow leopard scat samples was done by testing for the presence of the Y chromosome using primers that amplified an intron of the AMELY gene (i.e., a gene only found on the Y chromosome). The size of this fragment (Y chromosome) is about 200bp^24^.

A PCR reaction of the total volume of 7 μl was prepared containing 3.5 μl of Qiagen 2 × Master Mix Buffer, 0.7 μl of 5X Q solution, 0.05 μl of primers (AMELY-F and AMELY-R) 20 μM, 0.7 μl of distilled water, to which 2 μl of extracted undiluted DNA was added. The PCR reactions were done using the following thermocycling conditions: 95 °C for 15 min; followed by 45 cycles of each 94 °C for 15 s, 55 °C for 30 s and 72 °C for 1 min, with a final extension of 72 °C for 10 min. PCR was run in triplicate; a positive and negative control was maintained. The PCR products were run on a 2% agarose gel, stained with ethidium bromide and visualized under ultraviolet light. The PCR was done in triplicate (3 for each sample), three or two out of three male positives were considered positive. One of three male positives was always repeated to re-confirm the identity. If the repeats were 1/3 for each triplicate, they were considered male based on 6 replicates. Only 3 of 3 negatives were considered to be female. If only one out of six replicates revealed Y chromosome, the samples were regarded as of unconfirmed sex.

### Microsatellite based Individual ID

A set of 6 microsatellite loci located on 6 different chromosomes of snow leopard was targeted using sets of the following six fluorescent dye tagged primers in two combinations (Table 2). Six polymorphic microsatellite loci chosen were sufficient to give the value of PID (Probability of Identity) that was adequate to characterize individuals in a population sharing the same genotype^24^.

**Table 2:** Six microsatellite loci used for determining the Individual ID of snow leopards (according to Janecka et al.^24^ and Rozhnov et al.^57^).

Locus name	Repeat motif	Size range (bp)	Chromosome	Label
PUN1157	(AC)17	101–109	B3	Blue
PUN229	(GT)23	104–112	A1	Green
PUN124	(AC)22	90–100	A2	Black
PUN935	NA	110–120	D1	Blue
PUN894	(GT)17	110–118	C2	Green
PUN132	(GT)19	117–123	D3	Black

PCR reaction was done using a volume of 7 μl composed of 3.5 μl of Qiagen 2X Master Mix buffer, 0.7 μl of 5X Q solution and 2 μl of extracted DNA. 0.88 μl of 20 μM primers were used in the first combination, 0.78 μl of 20 μM primers and 0.02 μl of distilled water were used in the second combination. A multiple tube approach^58^ was used for the multiple PCR reactions of each sample, in this case three times. PCR product from both combinations was further diluted to 1:50 and processed using an ABI 31 Analyzer. Three replicates of all the samples were processed.

### Genetic analysis and population structure

After processing the 6-microsatellite based PCR product through capillary electrophoresis using an ABI 31 Analyzer, the labelled fragments by size were separated and retrieved in .fsa files. All these .fsa data files were analysed using different software to assign the allele calls, to determine the genetic diversity and to describe the population genetic structure by Bayesian clustering in Structure (see Benešová^37^ for details). Allele calling was done using GeneMarker V1.85 (https://www.softgenetics.com), then consensus genotypes were reconstructed and values adjusted using Autobin software (https://www6.bordeauxaquitaine.inra.fr/biogeco_eng-/Scientific-Production/Computer-software/Autobin). Presence of genotyping errors due to potential artefacts such as a large allele drop out, stuttering bands etc. was estimated using Micro-Checker^59^.

Occurrence of identical genotypes was analysed using the software Cervus 3.0.7^60^. Basic descriptive parameters of the population genetics were computed using GeneAlEx 6.5^61^. Population genetic structure was described using Bayesian clustering and software Structure 2.3.4^62,63^ assuming the clusters differ relative to each other in allele frequency. An analysis was made of the most probable number of clusters and the probability of assigning individual samples to these clusters^63^. Models that presume correlated allelic frequencies and incorporate a biogeographical ancestry analysis were used^64^. Five iterations for each K with 10^6^ MCMC steps and burn-in set to 10^5^ were performed. The possible number of populations was set in the range of 1 to 10 and for each possible number of populations there were 5 runs. Results were visualized using Structure Selector^65^, the most probable number of clusters was determined using parameter ΔK^66^ and the values for each K were combined in Clumpp 1.1.2^67^.

### Occurrence records of snow leopard

Data on occurrence, including camera trapping and GPS locations of genotyped scats, were collected at the altitudinal range between 3000 – 5200 m during 2014 – 2016. For snow leopard monitoring, camera traps Bushnell HD cameras with passive infrared detector Trophy Camera were used. Their numbers varied between years: the minimum was 32, the maximum was 48 cameras. Thus, we have 4567 trap nights covering two trapping sessions per season (wet and dry seasons) at each site during 2014–2016. Some opportunistic records of scrapes of snow leopards were also collected during 2004 – 2016 in the regions studied and based on these a habitat suitability map was produced. There were 628 records of occurrence at 482 different locations, mostly from Sagarmatha National Park and Annapurna Conservation Area (particularly Upper Manang and Lower Mustang).

### Habitat suitability and connectivity analyses

Models of habitat suitability indicate both the actual and potential occurrence of a focal species^68^. Beside the definition of suitable or stepping stone areas important for dispersal or migration, outputs of habitat suitability models were used for the construction of resistance surfaces. Inputs needed for habitat suitability models are data on the occurrence of the focal species and relevant environmental variables.

Based on species–habitat associations method by following Gavashelishvili and Lukarevskiy^69^ for description of the environmental conditions and preparation of the habitat suitability model, habitat variables related to terrain (topography), climate, habitat (land cover) and effect of human activity (distance to nearest roads) were used based on the published papers^7,15,34,44,53^. The field data were obtained from the Shuttle Radar Topography Mission (SRTM) elevation data and these data were also used to calculate the potential annual solar radiation (Megajoule (mj) cm^−2^ year^−1^), following Gavashelishvili and Lukarevskiy^69^. The ruggedness data was obtained following Sappington et al.^70^ and Riordan et al.^21^. The ruggedness index in the output raster can range from 0 (no terrain variation) to 1 (complete terrain variation). All these layers of environmental variables were prepared in ArcMap 10.6.1. All data were converted into a uniform raster with a 100-m spatial resolution in ASCII format. Habitat analysis and potential distribution modelling were run using Maxent 3.4.1^38,39^ and 25% of the occurrence data was used to verify the model. Results of the Maxent model were verified by ROC values whereas models having AUC scores > 0.75 were considered potentially useful and indicate excellent predictive ability^38,39^.

Based on pilot studies (not presented here), the set of best-performing environmental variables was selected, which was then used in the final model. These variables are presented in Table 3.

**Table 3:** Environmental variables used in the habitat suitability model. Those that were most closely associated with the presence of snow leopard in the region studied are in bold.

	Variable	Source
Topography	Altitude	SRTM (© CGIAR-CSI, 2004)
	Ruggedness index	SRTM (© CGIAR-CSI, 2004)
	Solar radiation	SRTM (© CGIAR-CSI, 2004)
Climate	Annual mean temperature	WorldClim 2.0 (Fick et al. 2017)
	Mean diurnal range of temperatures	WorldClim 2.0 (Fick et al. 2017)
	Annual precipitation	WorldClim 2.0 (Fick et al. 2017)
Habitat	Land cover	FAO Global Land Cover Network
Effect of human activity	Distance to roads	Open Street Maps (2017)

Inverse raster of habitat suitability provided a resistance surface enabling a further analysis of connectivity. Euclidean distances between localities were calculated using a Geographic Distance Matrix Generator 1.2.3^71^. Landscape connectivity and resistance distances in the area modelled were analysed using the circuitscape theory^72^. Software Circuitscape 4.0^73^ generated resistance distances between all the localities of the genetic samples, which is the most important output for further integration of geographical and genetic methods. At the same time, voltage maps showing the most probable direction in which individuals moved and consequently the directions of gene flow were produced. The interconnection of the patches where the habitat was suitable for snow leopard was obtained from the habitat model. The most suitable areas were then defined as those for which the habitat model predicts suitability index > 0.5 and an area of at least 10 km^2^.

### Landscape genetic approach and data integration

To assess the spatial structure of the population, we used the landscape genetic approach^74^. To analyse the role of particular environmental factors in the spatial ecology of snow leopard, isolation by distance (IBD)^75^ and isolation by resistance (IBR)^76,77^ were used. Genetic distance measured as F_ST_^76^ was obtained from Genepop 4.2^78,79^. The genetic distance matrix was compared with the geographic and resistance distance matrices using the Mantel test^80^ in PASSaGE 2.0 software^81^.

### Ethical approval

The research permit was approved by Department and National Parks and Wildlife Conservation and National Trust for Nature Conservation of Nepal.

## Data Availability

Occurrence locations of snow leopards cannot be presented here due to risk of poaching. We can provide the data to individual researchers upon formal request to either of the authors.
